# Integrating machine learning interatomic potentials with hybrid reverse Monte Carlo structure refinements in *RMCProfile*

**DOI:** 10.1107/S1600576724009282

**Published:** 2024-10-29

**Authors:** Paul Cuillier, Matthew G. Tucker, Yuanpeng Zhang

**Affiliations:** a Department of Materials Science and Engineering The Ohio State University Columbus OH43212 USA; b Neutron Science Division Oak Ridge National Laboratory Oak Ridge TN37831 USA; Australian Synchrotron, ANSTO, Australia

**Keywords:** reverse Monte Carlo, machine learning, interatomic potentials, total scattering

## Abstract

Structure refinement with reverse Monte Carlo (RMC) is a powerful tool for interpreting experimental diffraction data. To ensure that the under-constrained RMC algorithm yields reasonable results, the hybrid RMC approach applies interatomic potentials to obtain solutions that are both physically sensible and in agreement with experiment. To expand the range of materials that can be studied with hybrid RMC, we have implemented a new interatomic potential constraint in *RMCProfile* that grants flexibility to apply potentials supported by the *Large-scale Atomic/Molecular Massively Parallel Simulator* (*LAMMPS*) molecular dynamics code. This includes machine learning interatomic potentials, which provide a pathway to applying hybrid RMC to materials without currently available interatomic potentials. To this end, we present a methodology to use RMC to train machine learning interatomic potentials for hybrid RMC applications.

## Abstract 2

New software capabilities in *RMCProfile* allow researchers to study the structure of materials by combining machine learning interatomic potentials and reverse Monte Carlo.

## 1. Introduction

The first step to understanding fundamental material structure–property relationships is understanding the structure. For disordered materials, the X-ray and neutron total scattering techniques simultaneously probe long- and short-range order by considering both Bragg and diffuse scattering information (Egami & Billinge, 2012 ▸). These reciprocal-space diffraction measurements are often more easily interpreted in real space by applying Fourier transformations to generate pair distribution functions (PDFs), which represent histograms of interatomic distances within a material. It is straightforward to simulate a diffraction pattern and PDF from an atomistic model of a structure, so it is natural to ask the inverse question – what structure could have produced the observed diffraction pattern? This can be answered using the reverse Monte Carlo (RMC) method, which aims to derive an atomistic model that fits experimental data (McGreevy & Pusztai, 1988 ▸).

Over many iterations of stochastically displacing or swapping atoms, moves that improve the agreement between the simulated and experimental diffraction patterns are accepted to drive the model towards a structure consistent with experimental data. This is analogous to Metropolis Monte Carlo (MMC), except the model is driven by a χ^2^ measure of the fit quality instead of the system energy (Metropolis *et al.*, 1953 ▸). To co-refine a model against multiple experimental measurements, the RMC χ^2^ can include contributions from a combination of X-ray and neutron total scattering (McGreevy *et al.*, 1992 ▸), Bragg diffraction (Tucker *et al.*, 2001 ▸), extended X-ray absorption fine structure (Krayzman *et al.*, 2009 ▸), single-crystal diffuse scattering (Krayzman & Levin, 2012 ▸) and fluctuation microscopy (Maldonis *et al.*, 2017 ▸).

In practice, it is rarely possible to uniquely determine the three-dimensional structure of a material from diffraction data alone. This can be attributed to multiple factors, including weakly scattering elements, instrument resolution limitations (Zhang *et al.*, 2020 ▸), under-constrained partial radial distribution functions (Soper, 1996 ▸) and many-body interactions between atoms that are not directly measurable with pair (two-body) distribution functions (McGreevy & Howe, 1991 ▸; Howe *et al.*, 1993 ▸). When combined with the inherently stochastic Monte Carlo algorithm, this uniqueness problem can lead RMC to yield excessively disordered structures. To prevent unphysical arrangements of atoms, additional restraints like bond distance limits, target coordination numbers or bond valence sums can be applied. However, identifying certain arrangements of atoms as unphysical and preventing them with *ad* *hoc* restraints requires chemical intuition and RMC expertise from the practitioner.

An alternative solution is to combine the RMC and MMC approaches into hybrid RMC (HRMC), where the energy of the system is considered in the RMC χ^2^ to quantitatively discourage unphysical arrangements of atoms (Opletal *et al.*, 2008 ▸). Ideally, this yields structures that are both physically sensible and in good agreement with experiment. The barrier to using this method is that one needs a fast and accurate interatomic potential to calculate the energy for different arrangements of atoms. For this reason, HRMC has primarily been applied to material systems that benefit from decades of potential development, such as disordered carbons (Jain *et al.*, 2006 ▸; Farmahini & Bhatia, 2015 ▸), metallic glasses (Hwang *et al.*, 2012 ▸), and some oxides and carbides (Khadka *et al.*, 2020 ▸; Pandey *et al.*, 2015 ▸). Likewise, RMC software packages that currently support HRMC are oriented towards these usually non-crystalline materials (Gereben *et al.*, 2007 ▸; Gereben & Pusztai, 2012 ▸; Opletal *et al.*, 2014 ▸). For polycrystalline materials and systems without potentials compatible with existing RMC codes, the technical barrier to leveraging HRMC has been prohibitively high.

To enable HRMC for a wider variety of materials, we have expanded the interatomic potential constraints in *RMCProfile* (Tucker *et al.*, 2007 ▸; Zhang *et al.*, 2020 ▸), the most developed RMC framework for polycrystalline materials. The new potential constraint leverages the *Large-scale Atomic/Mol­ecular Massively Parallel Simulator* (*LAMMPS*) code (Thompson *et al.*, 2022 ▸) to calculate the energy, allowing users to utilize a wider range of interatomic potentials, including many archived in databases like the NIST Interatomic Potentials Repository (Hale *et al.*, 2018 ▸; Becker *et al.*, 2013 ▸) and OpenKIM (Tadmor *et al.*, 2011 ▸). For material systems without a currently available potential, *LAMMPS* also enables the use of highly accurate, material-specific machine learning interatomic potentials (MLIPs). By encoding the local atomic structure into a high-dimensional descriptor, a machine learning model can accurately reproduce energies, forces and stresses from reference density functional theory (DFT) calculations. However, like many machine learning models, MLIPs can be poor at extrapolation, so they should be trained on structures that cover the bounds of possible local environments that the MLIP needs to reliably describe. This makes RMC, which inherently tends to sample the most disordered local environments consistent with experimental data, a promising method of generating robust training data for MLIPs. We demonstrate how the statistical nature of RMC can be leveraged to generate diverse training structures for an MLIP, which, in turn, can be applied to reinforce confidence in interpretations of experimental data through HRMC refinements.

## 2. *LAMMPS* potential energy constraint in *RMCProfile*

### 2.1. Implementation

To refine an atomistic model consistent with experiment, RMC uses a Monte Carlo algorithm to minimize a χ^2^ measure of the difference between the experimental and calculated data patterns (McGreevy & Pusztai, 1988 ▸; Tucker *et al.*, 2007 ▸). To optimize fits to multiple data sets simultaneously, the total 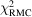 is composed of contributions from individual 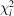 values weighted by factors σ_*i*_ to balance the relative significance of each constraint:
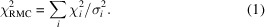


On each iteration, an atom is randomly displaced or swapped with another atom, and the change in the overall quality of the fit 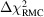 is calculated. If a move improves the fit (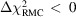), it is accepted. Otherwise, it is accepted with probability 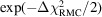 to allow some number of ‘bad’ moves that may be necessary to escape from local minima.

Like the harmonic bond stretching and bending potential constraints previously implemented in *RMCProfile*, the new interatomic potential constraint penalizes energetically unfavorable moves by adding the MMC term 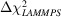 = 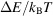 to the total 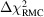, where Δ*E* is the change in energy from the proposed move, normalized by the product of temperature *T* and Boltzmann constant *k*_B_. The new potential constraint calculates Δ*E* by interfacing with *LAMMPS* to expand the range of potentials that can be used with *RMCProfile*. Currently, the only restriction is that the potential must be purely local, meaning atoms only interact within some short (usually <10 Å) cutoff radius. This way, only the region around the moved atom needs to be considered in the energy calculation. For typical RMC supercell sizes of >1000 atoms, this approximation allows the computational cost of the potential constraint to remain small compared with the normal RMC routines. However, it introduces a trade-off between the number of atoms considered in the calculation and the accuracy in the calculation of Δ*E* and 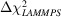. This is discussed in more detail in Appendix *B*[App appb]. For most traditional interatomic potentials and descriptor-based MLIPs, this local assumption is reasonable. Some non-local exceptions that would be unsuitable as *RMCProfile* constraints are graph neural network MLIPs and potentials that handle electrostatic interactions in reciprocal space. This local requirement is only necessitated by computational limitations, which may be overcome in the future.

## 3. Application: oxygen vacancy ordering in CeO_2−*x*_

### 3.1. Background

**Figure 1:**
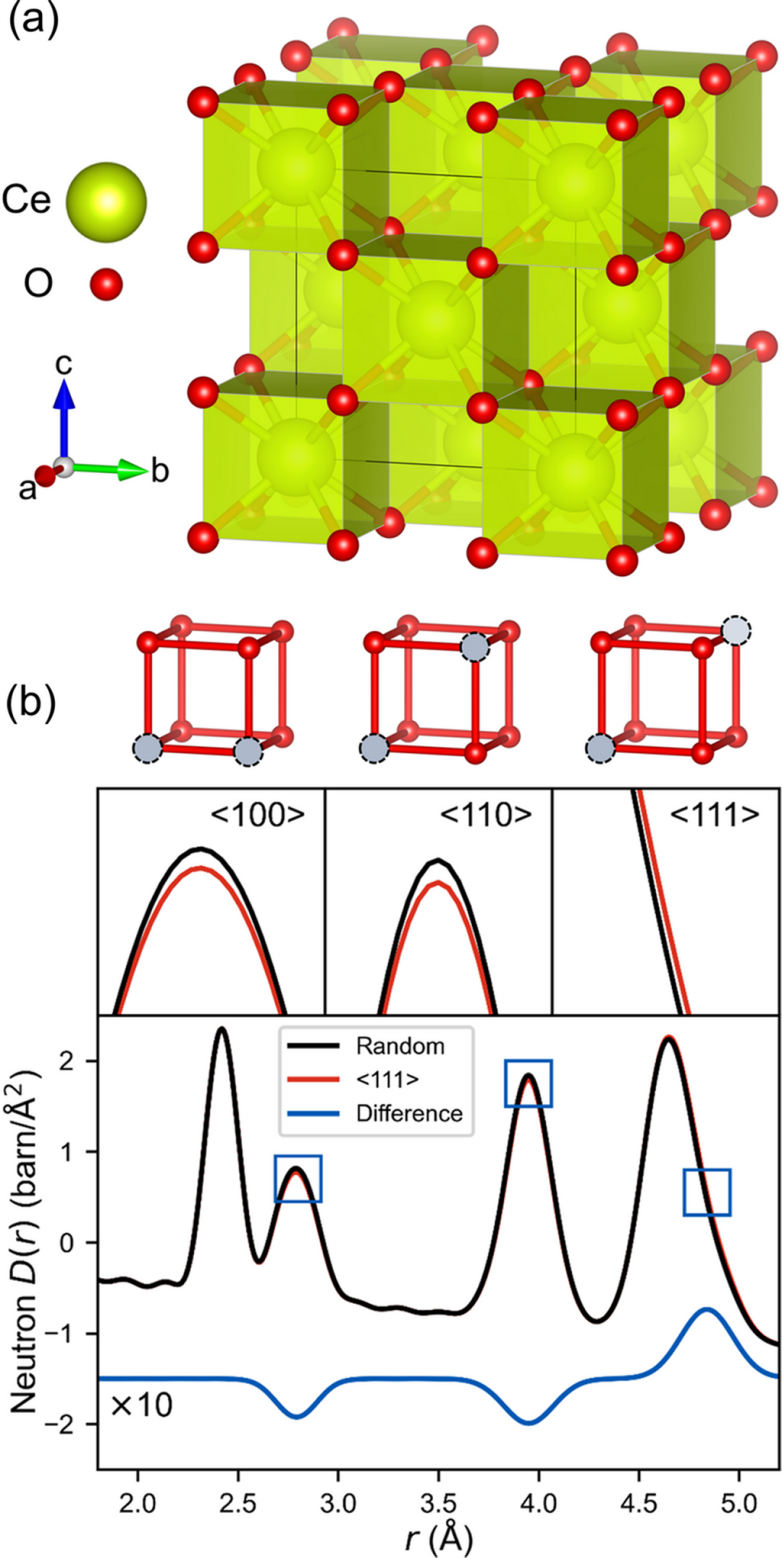
(*a*) Fluorite average structure of ceria. (*b*) Simulated neutron PDF for CeO_1.71_ with a random vacancy distribution (black) compared with the same composition with all vacancy pairs ordered along 〈111〉 (red). The ordering results in slight differences (blue, ×10 magnification) in the intensities of peaks from O–O interatomic distances.

To illustrate an application of the new potential constraint, we apply it to the study of oxygen vacancy ordering in partially reduced ceria (CeO_2−*x*_). Cerium(IV) oxide has a fluorite structure (space group 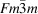), shown in Fig. 1 ▸(*a*), with a face-centered cubic arrangement of cerium atoms and oxygen atoms occupying tetrahedral holes. The fluorite phase can accommodate a significant number of oxygen vacancies within concentrations between CeO_2_ and ∼CeO_1.7_ (Panlener *et al.*, 1975 ▸; Hull *et al.*, 2009 ▸). While Bragg diffraction analysis of the average structure suggests a random distribution of vacancies, DFT studies predict that pairs of vacancies order along certain crystallographic directions, avoiding nearest-neighbor sites in favor of 〈110〉 and 〈111〉 chains (Murgida *et al.*, 2014 ▸). Because this short-range oxygen vacancy ordering changes the number of O–O pairs at various interatomic distances, it can be probed by neutron total scattering and PDF analysis. In Fig. 1 ▸(*b*), the effect is simulated for CeO_1.71_ with vacancies ordered along 〈111〉 chains and compared with a random vacancy distribution. While the effect of vacancy ordering on the neutron PDF is small, it is significant enough that careful RMC analysis has previously corroborated the prediction that the nearest-neighbor 〈100〉 ordering is less stable than the 〈110〉 and 〈111〉 orderings (Hull *et al.*, 2009 ▸). Since this phenomenon is a result of many-body interactions, we use it to demonstrate how the new potential constraint can be leveraged to make RMC refinements more sensitive to structural features that are otherwise challenging to study with experimental data alone.

### 3.2. RMC refinements

To isolate the effect of the potential constraint, we apply it to RMC refinements of simulated X-ray and neutron PDF data. This way, the ground-truth structure that RMC aims to reproduce and the underlying interatomic interactions are known. In this case, the underlying physics of the system is defined by a ReaxFF potential designed for CeO_2_ nanoparticles and partially reduced CeO_2−*x*_ (Broqvist *et al.*, 2015 ▸). Ground-truth structures were obtained from constant *NVT* molecular dynamics (MD) simulations, using an 8 × 8 × 8 supercell of the CeO_1.71_ fluorite average structure from Hull *et al.* (2009 ▸; Inorganic Crystal Structure Database 246972). To redistribute oxygen vacancies, the structure was equilibrated at 1273 K for 50 ps, then quenched to 100 K over 10 ps before taking a snapshot of the MD simulation. This annealing cycle was repeated 30 times to obtain structures with independent vacancy distributions. The X-ray and neutron total scattering data used in the RMC fitting were obtained by averaging the calculated patterns for the 30 MD snapshots, using a *Q*_max_ of 20 and 30 Å^−1^ for X-ray and neutron data, respectively. Representations of the total scattering data were fitted in reciprocal space to the structure function *F*(*Q*) = *S*(*Q*) − 1 and in real space to the differential correlation function 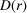 = 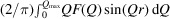, defined according to the *RMCProfile* convention (Keen, 2001 ▸; Tucker *et al.*, 2007 ▸; Peterson *et al.*, 2021 ▸). The automatic weight optimization routine (Zhang *et al.*, 2020 ▸) was used in the RMC fitting, so minimum 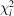 limits for each constraint were set to the average 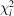 values across the 30 MD snapshots. The initial atomic configuration was an 8 × 8 × 8 supercell of the fluorite unit cell (6144 atoms), with randomly distributed oxygen vacancies. To allow the RMC algorithm to refine the oxygen vacancy ordering, every fifth proposed RMC move swapped an oxygen to a vacant site.

### 3.3. RMC refinements of simulated data

**Figure 2:**
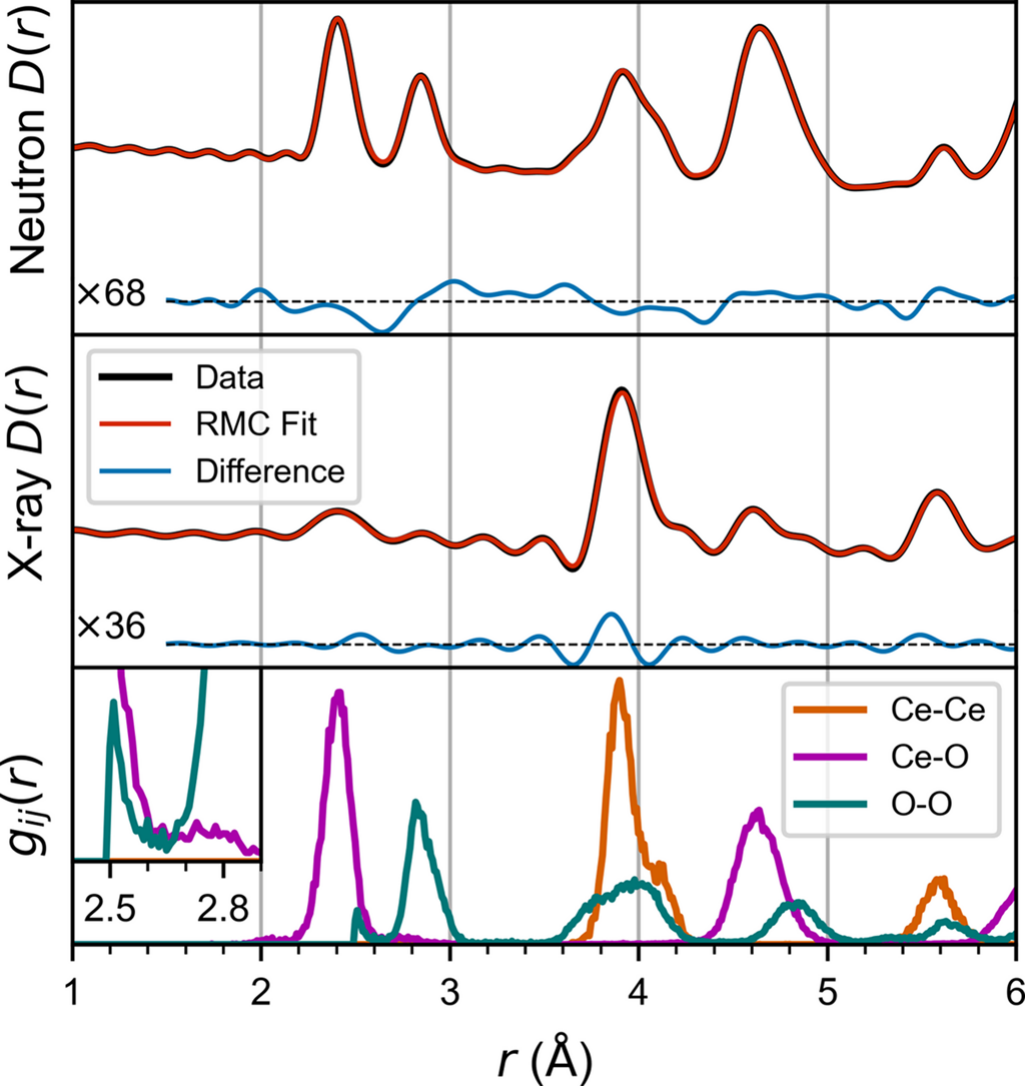
RMC refinement with simulated neutron and X-ray*D*(*r*) data constraints, with difference curves drawn at 68× and 36× magnification, respectively. The partial radial distribution functions *g*_*ij*_(*r*) are shown in the bottom panel, with an inset to magnify the region where unphysical features appear in the Ce–O and O–O radial distribution functions.

**Figure 3:**
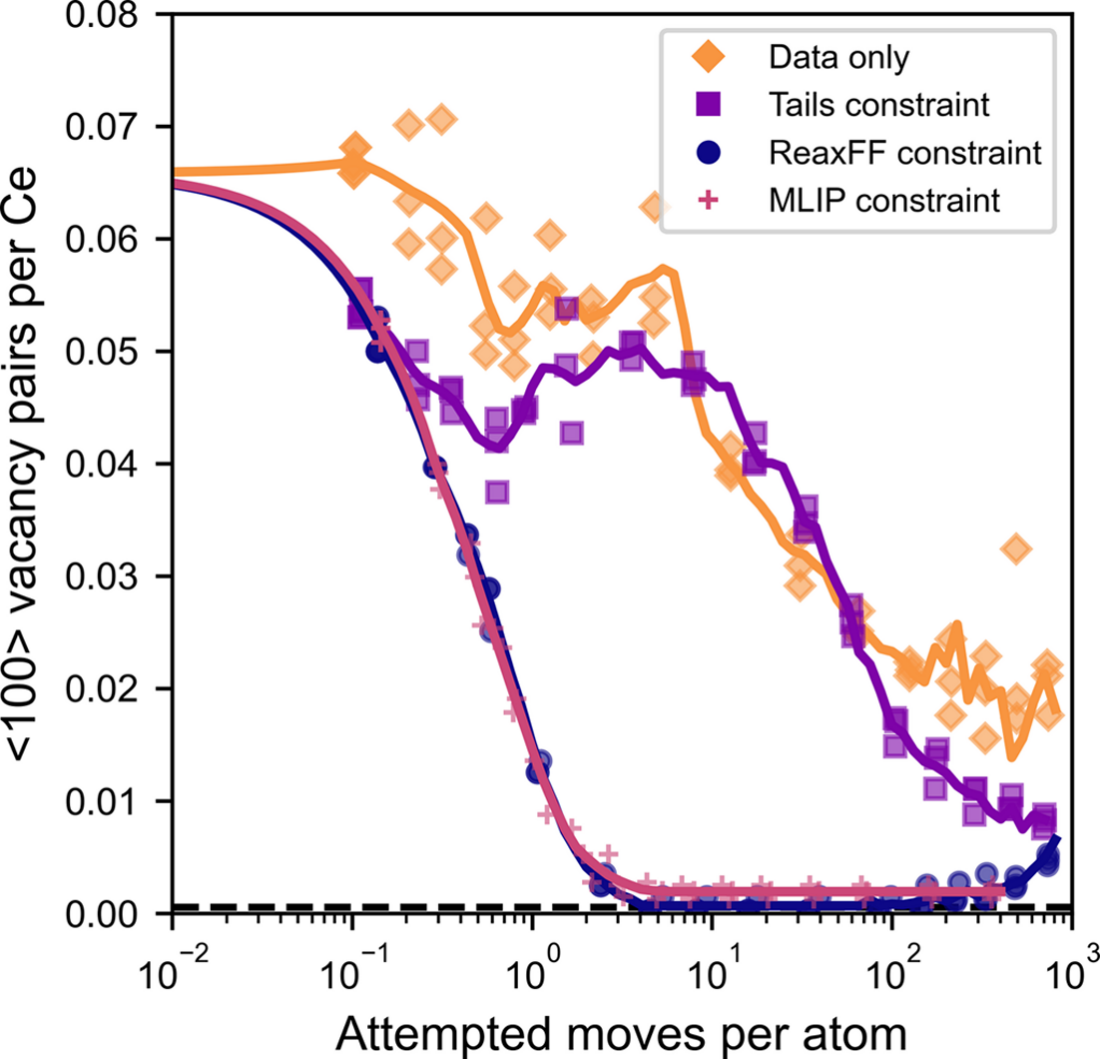
Convergence of the concentration of 〈100〉 vacancy pairs over the course of RMC refinements with and without potential constraints. The dashed line near zero marks the target vacancy concentration in the ground-truth structures.

This RMC refinement of simulated data represents a case with perfect data reduction procedures and instrument corrections. Even so, with only X-ray and neutron total scattering data constraints, there are unphysical features due to the under-constrained nature of RMC. In this case, it arises because only two measurements are used to constrain three partial radial distribution functions for Ce–Ce, Ce–O and O–O pair correlations (Soper, 1996 ▸). As a result, the RMC algorithm can fit the simulated data by cross-contaminating the tails of peaks in the partial radial distribution functions, where the Ce–O and O–O nearest-neighbor distances overlap around 2.5 Å. This can be seen in the bottom panel of Fig. 2 ▸, where there is an accumulation of O–O pairs at the 2.5 Å minimum distance. This is a common unphysical feature in RMC refinements, but whether it significantly influences conclusions of the RMC analysis depends on the structural feature of interest. Here, the question is whether the RMC refinement can reproduce the ground-truth oxygen vacancy ordering, which is represented by the number of 〈100〉 vacancy pairs in Fig. 3 ▸. In this case, because the vacancy ordering is derived from the number of O–O correlations at each distance (*i.e.* the area of each peak), this unphysical mixing of the Ce–O and O–O partials prevents the RMC refinement from reaching the correct ordering with nearly zero 〈100〉 vacancy pairs.

In *RMCProfile*, these unphysical features can be addressed *ad* *hoc* by constraining the tails of the Ce–O and O–O partials to lie below user-defined sigmoid functions. In practice, the exact choice of sigmoid function parameters will influence the area of the O–O peak, so the result is still somewhat dependent on intuition. To consider the best-case scenario for the tail constraint, the sigmoid parameters were obtained from the true partial radial distribution functions, which are only known because the data are simulated. Application of this constraint yields a slight improvement, shown in Fig. 3 ▸. With appropriately chosen tail constraint functions, the RMC refinement slowly converges towards the correct 〈100〉 vacancy pair concentration.

**Figure 4:**
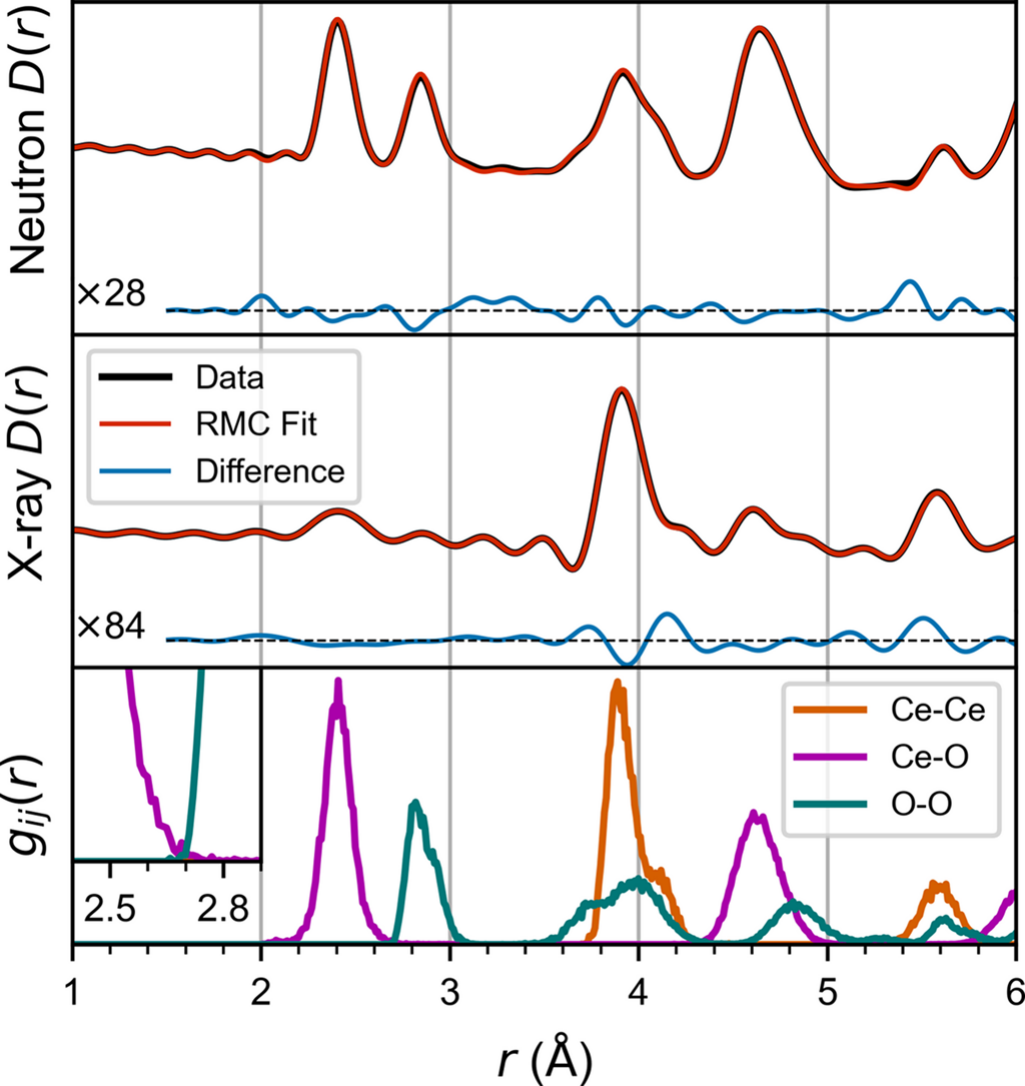
RMC refinement with simulated neutron and X-ray*D*(*r*) data and a ReaxFF potential constraint, with difference curves drawn at 28× and 84× magnification, respectively. The partial radial distribution functions *g*_*ij*_(*r*) are shown in the bottom panel.

The slow convergence of the vacancy ordering can be attributed to the fact that it is a result of many-body interactions, but the experimental PDF data only explicitly contain information about two-body correlations. The effect of vacancy ordering on the experimental data is small (see Fig. 1 ▸), but the energetic penalty of creating a 〈100〉 vacancy pair is large, on the order of several hundred meV (Murgida *et al.*, 2014 ▸). With the expanded capability to use many-body potentials as constraints, this information can be considered in the RMC refinement. When the same ReaxFF potential used to generate the simulated data is applied to constrain the RMC fitting, it correctly eliminates the 〈100〉 vacancy pairs in 100 times fewer attempted moves. This is shown in Fig. 3 ▸ for both the ground-truth ReaxFF potential and the MLIP trained to reproduce it. As shown in Fig. 4 ▸, the HRMC refinement yields a satisfactory fit to the simulated data, while eliminating the unstable 〈100〉 vacancy pairs and the unphysical features in partial radial distribution functions. This illustrates the utility of the expanded potential constraint in *RMCProfile*.

## 4. RMC-trained machine learning interatomic potentials

In this example, the ReaxFF potential used as an RMC constraint captures the true physics of the system only because it was used to generate the ground-truth structures from which the diffraction patterns were simulated. In practice, there is rarely an interatomic potential available for the specific material system of interest, let alone one with perfect agreement with experiment. Fortunately, it has recently become more feasible to develop highly accurate MLIPs trained on DFT calculations. By using RMC to generate training data for an MLIP, the results of the ReaxFF constraint in Figs. 3 ▸ and 4 ▸ can be closely reproduced, which is encouraging for the prospects of applying HRMC to new material systems. This section discusses a methodology for training MLIPs suitable for HRMC refinements.

### 4.1. Machine learning interatomic potentials

For many atomistic simulations, one needs to calculate the energy and its derivatives (forces and stresses) for some arrangement of atoms. Ideally, these would be calculated from first principles with DFT. Unfortunately, the nonlinear scaling of electronic structure optimization methods makes this computationally impractical for applications like HRMC, which require many calculations with more than several hundred atoms. For these situations, the electronic structure calculation must be circumvented by treating the interactions between atoms classically, as parameterized by an interatomic potential. In recent years, it has become more common to use MLIPs as surrogates for expensive DFT force calculations (Deringer *et al.*, 2019 ▸; Behler, 2016 ▸). This is often accomplished by representing atomic positions as a high-dimensional descriptor vector, which functions as the input to a machine learning model trained to reproduce DFT-calculated energies, forces and stresses for different structures. Numerous combinations of descriptors and machine learning frameworks have been demonstrated to be effective, such as deep neural networks (Zhang *et al.*, 2018 ▸), moment tensor potentials (Shapeev, 2016 ▸), spectral neighbor analysis potentials (Thompson *et al.*, 2015 ▸), atomic cluster expansions (Drautz, 2019 ▸) and Gaussian approximation potentials (Bartók & Csányi, 2015 ▸; Deringer *et al.*, 2021 ▸).

Regardless of which MLIP is used, it must be trained on a set of reference DFT calculations. An increasingly common method of obtaining the reference structures needed to train an MLIP is active learning, as it can significantly accelerate and streamline the process (Podryabinkin & Shapeev, 2017 ▸; Podryabinkin *et al.*, 2019 ▸; Jinnouchi *et al.*, 2020 ▸). The general approach involves sampling an atomic configuration space by proposing many possible structures of interest, but only performing expensive DFT calculations for ones that are significantly different from structures already represented in the training data. The method used to sample the atomic configuration space should be computationally inexpensive and capable of generating diverse structures that span the range of situations the MLIP needs to reliably describe. Common methods are genetic algorithms (McCall, 2005 ▸) and on-the-fly molecular dynamics (OTF MD) (Li *et al.*, 2015 ▸). After proposing many candidate structures, novel ones that warrant an expensive DFT calculation can be identified with uncertainty quantification, through Bayesian inference (Vandermause *et al.*, 2020 ▸; Deringer *et al.*, 2021 ▸), extrapolation grade determination (Lysogorskiy *et al.*, 2023 ▸), query by committee (Schran *et al.*, 2020 ▸) or any other uncertainty quantification method suitable for machine learning models (Zaverkin & Kästner, 2021 ▸; Abdar *et al.*, 2021 ▸). By limiting the number of redundant DFT calculations, these active learning approaches reduce the computational cost of training MLIPs and improve their reliability by sampling more diverse training structures (Chan *et al.*, 2019 ▸).

### 4.2. Training MLIPs on RMC results

RMC refinements generate an ensemble of local environments that fit experimental data in an average sense. In HRMC, the interatomic potential constrains RMC to a subset of these local environments that are also physically sensible. Thus, if the objective is to train an MLIP for HRMC, all possible local environments that the MLIP needs to describe should be represented in the original RMC results. For these purposes, RMC can serve the same function as genetic algorithms or OTF MD to generate candidate reference structures for training via active learning.

We demonstrate this by leveraging RMC to train a sparse Gaussian process regression (GPR) MLIP, using the *FLARE* package (Vandermause *et al.*, 2020 ▸; Xie *et al.*, 2021 ▸). This particular MLIP represents each atom’s local environment as an atomic cluster expansion descriptor vector (Drautz, 2019 ▸). To predict the forces acting on an atom and its contribution to the total energy, the GPR model quantifies the similarity of its local environment (as represented by its descriptor vector) to ones in the reference structures, using a kernel function like a dot product (Deringer *et al.*, 2021 ▸). The primary advantage of this kernel-based approach is that it enables uncertainty estimation, as predictions on a local environment that is far from any point in the training data should have higher uncertainty. *FLARE* accomplishes this with Bayesian inference, which allows fast and robust uncertainty quantification for active learning. To train an MLIP for HRMC, we use this uncertainty quantification to iteratively search RMC results for high-uncertainty local environments that warrant additional reference calculations.

**Figure 5:**
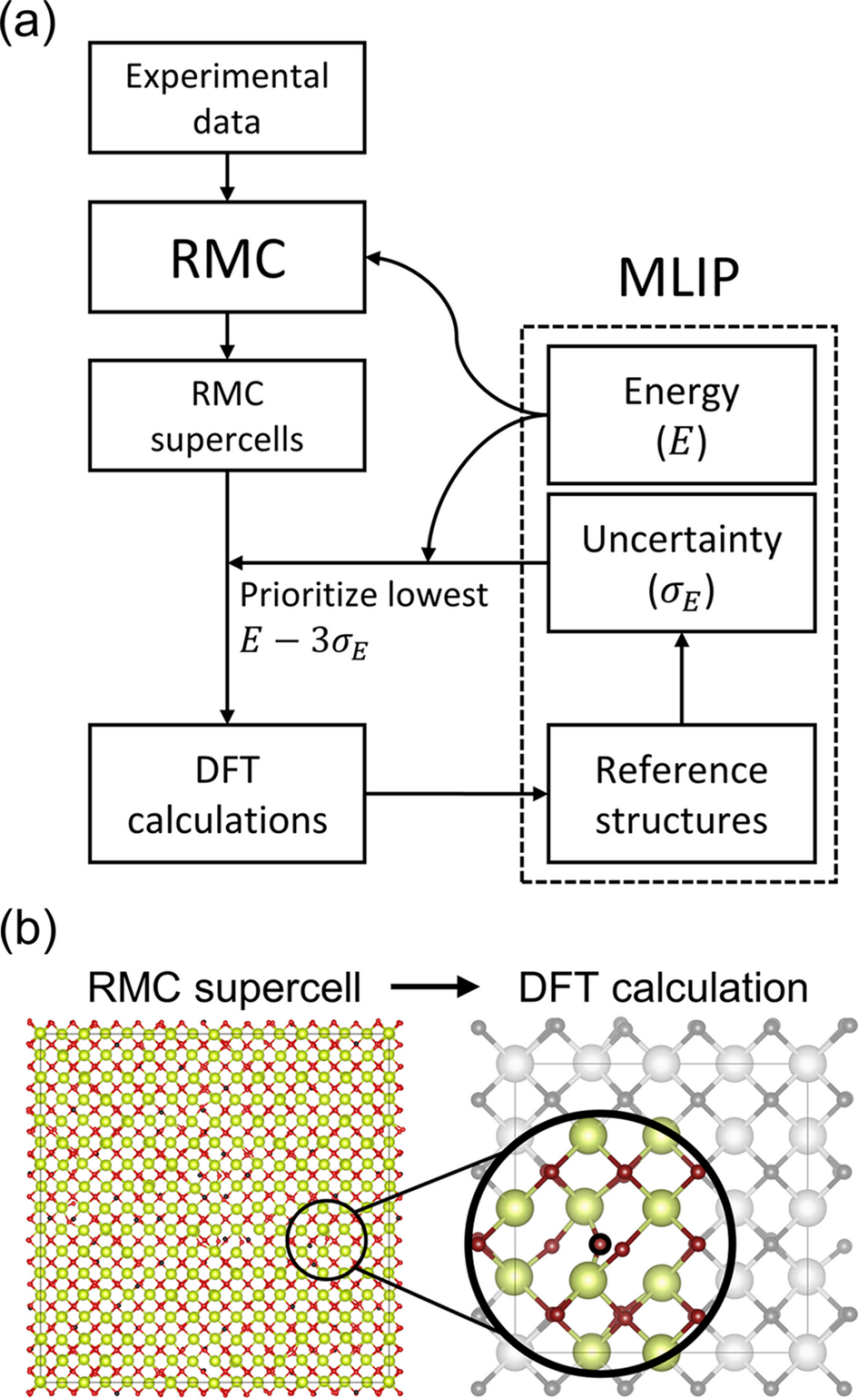
(*a*) Process flow for training MLIPs on results from RMC refinements, using an active learning approach. (*b*) Method for extracting a local environment from an RMC supercell and embedding it in a supercell suitable for DFT calculations. The colored atom positions are from the RMC supercell, within the cutoff radius of the high-uncertainty local environment. The remaining gray atoms are from the average positions.

The process of extracting training data from RMC results is outlined in Fig. 5 ▸(*a*). Starting from a small number of reference structures – in this case, the CeO_2_ ground state before and after small random displacements – the GPR model was used to quantify the uncertainty in the local energy contribution for each atom in the RMC supercells. For each high-uncertainty (>2%) local environment, a spherical cluster of atoms within the cutoff radius of the MLIP (5.2 Å) was extracted from the 8 × 8 × 8 RMC supercell and then embedded in a smaller 2 × 2 × 2 supercell that would be suitable for a DFT calculation [Fig. 5 ▸(*b*)]. The remaining atomic positions in the DFT-sized supercell were defined by the CeO_2_ average structure. This condensed ∼250000 local environments generated by accepted RMC moves down to ∼4500 candidate structures that would be novel additions to the reference data. Of these, candidates with both high uncertainty and low predicted energy were prioritized for reference calculations. While the diversity of the training data is important, excessively distorted structures are undesirable, because they increase the error of the final MLIP and may encounter DFT convergence issues (Miksch *et al.*, 2021 ▸). For these reasons, the candidate with the lowest 3σ confidence interval for the total cohesive energy was prioritized for a reference calculation. The selected candidate structure was used as the initial state of an on-the-fly damped dynamics geometry optimization, as used in other methods of training MLIPs (Choi & Jhi, 2020 ▸). The initial calculation and any required during the structural relaxation were added to the reference data set, the MLIP was retrained, and the energy predictions and uncertainties for the next 1000 highest-priority candidates were updated. Candidates without an atom in a high-uncertainty local environment were discarded, and the candidate with the lowest confidence interval for the cohesive energy (*E* − 3σ_E_) was selected for the next iteration. In this example, only 111 candidate structures, with a total of 208 reference calculations, were necessary to describe all candidates with energies <200 meV atom^−1^ above the ground state. The computational overhead from GPR predictions and retraining required a total of 35 core-hours, which is acceptably small compared with the thousands of core-hours expected for ∼200 accurate DFT calculations with ∼100 atoms. In this example, the ReaxFF potential provided the reference ground truth instead of DFT.

### 4.3. Validation of CeO_2_ MLIP

To compare the effectiveness of the RMC-based training method with other state-of-the-art methods, an MLIP was also trained with OTF MD (Xie *et al.*, 2021 ▸; Vandermause *et al.*, 2020 ▸). Constant *NPT* MD with a 2 × 2 × 2 CeO_2_ supercell with 8 vacancies was run at 500, 900, 1300 and 1700 K for 50 ps at each temperature. With the same uncertainty tolerance used in the RMC-based training, 864 reference calculations were required over the course of the OTF MD simulations. To avoid overfitting, the hyperparameters of the descriptor (interaction cutoff radius *r*_cut_ = 5.2 Å, number of radial basis functions *n*_radial_ = 10 and maximum angular momentum *l*_max_ = 4) and sparse GPR (dot product kernel variance σ = 5.3 eV, and energy/force/stress noise tolerances of σ_E_ = 2.1 eV atom^−1^, σ_F_ = 45 meV Å^−1^ and σ_S_ = 0.81 kbar) were determined by maximizing the marginal likelihood, which balances the accuracy and complexity of the GPR model (Deringer *et al.*, 2021 ▸; Vandermause *et al.*, 2022 ▸).

**Figure 6:**
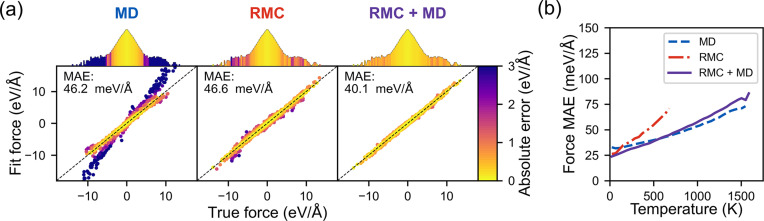
(*a*) Parity plots of force predictions on RMC structures, using different training data sets. From left to right, results are shown for the MLIP trained on reference structures generated with OTF MD, the RMC-based method presented here and the combined reference sets, respectively. The histogram above each parity plot shows the distribution of true forces on a logarithmic scale, with bin colors corresponding to the 95th percentile absolute error within each force range. (*b*) Mean absolute force error as a function of temperature for *NPT* MD up to 1750 K

To evaluate the suitability of the MD- and RMC-trained MLIPs as constraints for HRMC refinements, they were tested on 258500 local environments from RMC supercells collected over the course of refinements with only data constraints. As shown by the left parity plot in Fig. 6 ▸(*a*), the MD-trained MLIP systematically underestimates the stability of more distorted local environments that appear in the RMC results. If applied as a constraint in an HRMC refinement, it could introduce systematic errors in the RMC results by eliminating structural features that are necessary to fit the experimental data. If the stability were instead overestimated, the MLIP constraint could introduce unphysical features. In either case, it would be challenging to test this without some method of uncertainty quantification, because the ground-truth forces of >1000 atom RMC supercells cannot be calculated with DFT. To ensure reliable predictions when using an MLIP as a HRMC constraint, some of the MLIP reference structures should be derived from RMC results. The RMC-based training methodology eliminates the systematic errors, as confirmed by the center parity plot in Fig. 6 ▸(*a*). It also reliably predicts the interaction energies for pairs of vacancies, listed in Table 1 ▸. These determine the vacancy ordering, so it is important that the MLIP correctly predicts that 〈001〉 nearest-neighbor vacancy pairs are at least 300 meV higher energy than other orderings. For applications that only require a potential for HRMC refinements, this example suggests that the presented RMC-based training method can produce an accurate MLIP with relatively few reference DFT calculations.

This is not to imply that the RMC-based method is superior to state-of-the-art methods in general. Rather, they are complementary. The most accurate potential was obtained by combining the reference data sets from the RMC and OTF MD training methods, as shown by the right parity plot in Fig. 6 ▸(*a*). The purely RMC-trained MLIP is also less reliable when extrapolating to atomistic simulations beyond HRMC. This was tested for CeO_1.71_*NPT* MD simulations up to 1750 K, with the mean absolute force error as a function of temperature shown in Fig. 6 ▸(*b*). For the RMC-trained MLIP, the error quickly exceeded the force noise hyperparameter, before the structure unphysically melted at ∼600 K. The MD-trained MLIP did not encounter this issue. Combining the reference structure sets from the RMC- and MD-based training methods yielded an MLIP with accurate predictions for both RMC refinements and high-temperature MD simulations. For studies that might benefit from large-scale atomistic modeling beyond just HRMC, the RMC- and MD-based training procedures can be done in parallel.

## 5. Conclusions

We have implemented a new interatomic potential constraint in *RMCProfile* to enable HRMC structure refinements for a wider range of materials. The new constraint allows flexibility to use potentials supported by *LAMMPS*, including machine learning interatomic potentials for material systems without currently available alternatives. We illustrated the utility of the expanded HRMC capabilities for studying oxygen vacancy ordering in CeO_2_, where the potential drove the RMC refinement to the correct vacancy ordering and eliminated unphysical features without the need for additional *ad* *hoc* constraints. To accelerate interatomic potential development for new materials, we demonstrated an active learning approach that applied RMC to efficiently generate training data for MLIPs. We found these RMC-trained interatomic potentials are more reliable for HRMC purposes than potentials trained by other state-of-the-art means, but, as with all MLIPs, care should be taken when extrapolating to other atomistic simulations. For developing MLIPs that are sufficiently robust for both HRMC and other large-scale atomistic modeling, the RMC-based training method is a complementary addition to existing methods.

## Figures and Tables

**Figure 7:**
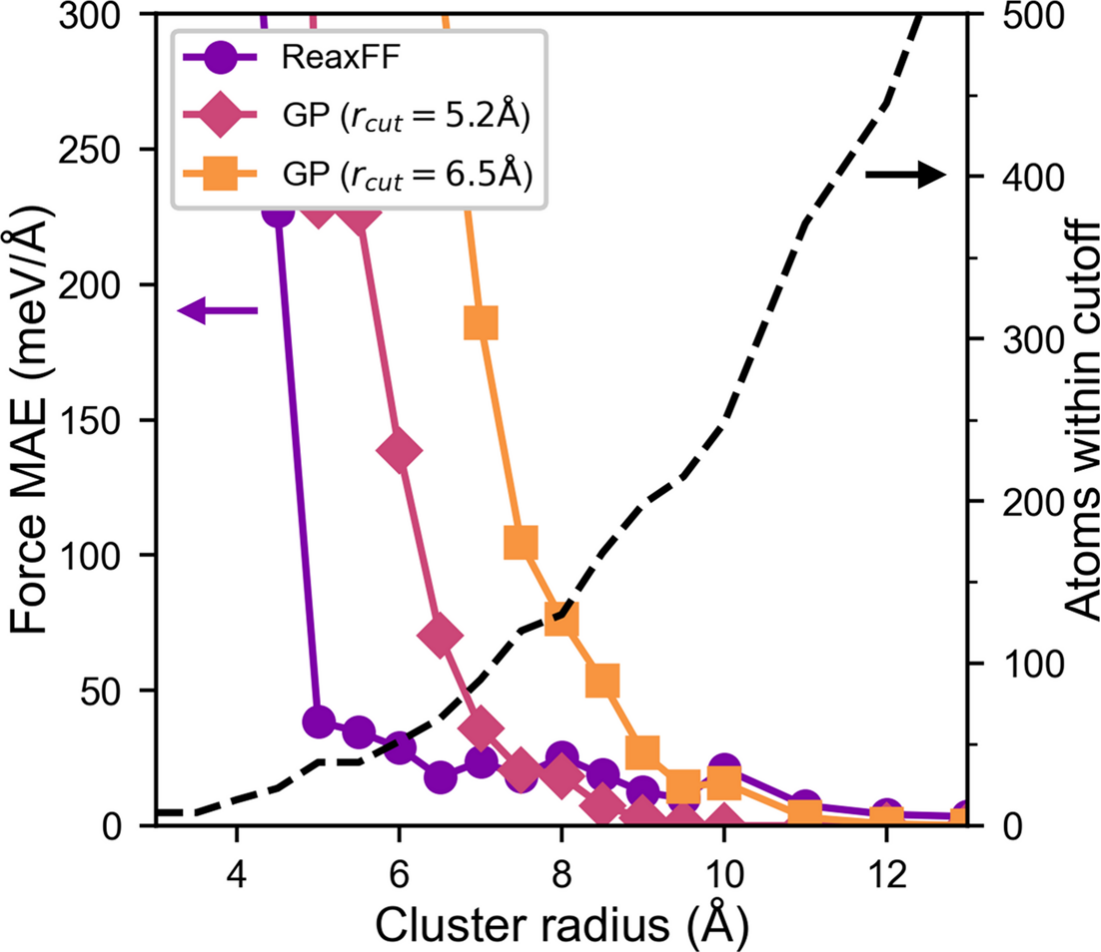
Error in force calculations as a function of the size of the cluster of atoms considered in the *RMCProfile* energy calculation. The computation time scales linearly with the number of atoms in the cluster (black dashed line).

**Table 1:** Relaxed interaction energies for pairs of vacancies (relative to isolated vacancies) at increasing separation distances within the oxygen sublattice Results are compared for the ground-truth ReaxFF potential and the RMC-trained MLIP.

Direction 〈*hkl*〉	Distance (Å)	*E*_ReaxFF_ (eV)	*E*_MLIP_ (eV)
〈100〉	2.73	0.584	0.541
〈110〉	3.87	0.117	0.108
〈111〉	4.74	0.235	0.161
〈200〉	5.47	0.301	0.313
〈210〉	6.12	0.001	0.012

## Appendix A Balancing potential and experimental constraint weights

In RMC refinements, the choice of weights for each constraint’s contribution to the total χ^2^ can significantly influence the resulting converged structure. This is especially true for energy-based constraints, where some energetically unfavorable ‘bad’ moves are always needed to account for thermal displacements. The weight of the potential constraint directly affects how many of these bad moves are accepted. If weighted too strongly, the convergence of the refinement will be slow and possibly confined to a local minimum. If weighted too weakly, the constraint will fail to prevent unphysical behavior. Finding a balance that yields a satisfactory fit and sensible partial radial distribution functions is somewhat subjective, so we find it advantageous to apply a range of potential constraint weights during the refinement. This is accomplished here with the weight optimization routine in *RMCProfile* (Zhang *et al.*, 2020 ▸), which adjusts weights on the fly to balance the relative importance of each constraint. To avoid overfitting, individual constraints can be turned off (weight set to zero) when their χ^2^ is below a specified minimum value. For the potential constraint, the minimum χ^2^ can be set to the expected total cohesive energy, causing the energy of the configuration to oscillate about this expected value. This is the weighting scheme used for the refinements in Figs. 3 ▸ and 4 ▸. The advantage of this approach is it only requires a single, physically justifiable parameter – the expected total energy. A possible disadvantage is that completely turning off the potential constraint may allow some number of unphysical moves. In the CeO_2_ example presented here, this results in an increase in the 〈100〉 vacancy pair concentration in the later stages of the ReaxFF-constrained refinement (see Fig. 3 ▸). These issues may be addressed by alternative weighting schemes, such as gradually increasing the weight of the potential constraint to perform a simulated annealing.

## Appendix B Locality assumption in *RMCProfile*

To minimize the computational cost of the potential constraint, it is assumed that the potential is purely local, such that atoms only interact within some cutoff radius *r*_cut_. In the new *RMCProfile* potential constraint, a spherical cluster of atoms within a distance *r*_cluster_ of the moved atom are given to *LAMMPS* to calculate the energy before and after the move. A smaller *r*_cluster_ will reduce the computational cost of the potential constraint, but an excessively small *r*_cluster_ will introduce errors in the energy calculation. For a two-body potential that only considers pairwise interactions within *r*_cut_, the local approximation only introduces errors for *r*_cluster_ < *r*_cut_. For potentials that include three-body and higher interactions, there may be errors for *r*_cluster_ < 2*r*_cut_ (Deringer *et al.*, 2021 ▸). If these errors cancel in the calculation of Δ*E*, a smaller *r*_cluster_ can still be used to reduce computation time. Fig. 7 ▸ demonstrates the error in the force calculation as a function of *r*_cluster_, for a CeO_2_ structure with a series of 1000 random 0.1 Å displacements, representative of typical RMC moves. The ReaxFF potential is a bond-order potential with short-range many-body interactions (Chenoweth *et al.*, 2008 ▸), so the error quickly decreases once *r*_cluster_ exceeds the next-nearest-neighbor Ce–O distance ∼4.5 Å. Small errors persist for high *r*_cluster_ values because of electrostatic interactions and a global charge equilibration routine with an effectively infinite interaction radius. In contrast, the GPR MLIPs consider five-body interactions within *r*_cut_. For *r*_cut_ = 5.2 Å, an *r*_cluster_ > 7 Å is needed to reduce the average force error below 50 meV Å^−1^ (approximately the MLIP force error hyperparameter). For *r*_cut_ = 6.5 Å, this requirement increases to *r*_cluster_ > 9 Å, which includes twice as many atoms and doubles the computational cost of the potential constraint. When selecting a potential to use as an RMC constraint, it will be most computationally efficient to use one with short-range many-body interactions like ReaxFF and embeded atom method potentials. When training a many-body MLIP specifically for refinements in *RMCProfile*, one should favor smaller *r*_cut_ when possible, as the typical improvement in accuracy with increasing *r*_cut_ is lost unless more atoms are included in the energy calculation. Hierarchical MLIPs that use a many-body machine learning descriptor for short-range interactions and two-body terms for long-range interactions may give the best compromise between accuracy and speed (Deringer *et al.*, 2021 ▸). In this CeO_2_ example with 5500 atoms (44.7 Å box dimensions), the ReaxFF constraint reduced the number of RMC moves per core-hour by a factor of ∼0.8, while the *FLARE* MLIP (with *r*_cut_ = 5.2 Å, *r*_cluster_ = 8.0 Å, *n*_radial_ = 10 and *l*_max_ = 4) reduced it by a factor of ∼0.1. Since the locality assumptions decouple the energy calculation time from the supercell size, the relative computational cost of the potential constraint will be less severe for larger supercells that have slower RMC routines.

## References

[bb1] Abdar, M., Pourpanah, F., Hussain, S., Rezazadegan, D., Liu, L., Ghavamzadeh, M., Fieguth, P., Cao, X., Khosravi, A., Acharya, U. R., Makarenkov, V. & Nahavandi, S. (2021). *Inf. Fusion*, **76**, 243–297.

[bb2] Bartók, A. P. & Csányi, G. (2015). *Int. J. Quantum Chem.* **115**, 1051–1057.

[bb3] Becker, C. A., Tavazza, F., Trautt, Z. T. & Buarque de Macedo, R. A. (2013). *Curr. Opin. Solid State Mater. Sci.* **17**, 277–283.

[bb4] Behler, J. (2016). *J. Chem. Phys.* **145**, 170901–170910. 10.1063/1.4971792 28799339

[bb5] Broqvist, P., Kullgren, J., Wolf, M. J., van Duin, A. C. T. & Hermansson, K. (2015). *J. Phys. Chem. C*, **119**, 13598–13609.

[bb6] Chan, H., Narayanan, B., Cherukara, M. J., Sen, F. G., Sasikumar, K., Gray, S. K., Chan, M. K. Y. & Sankaranarayanan, S. K. R. S. (2019). *J. Phys. Chem. C*, **123**, 6941–6957.

[bb7] Chenoweth, K., van Duin, A. C. T. & Goddard, W. A. (2008). *J. Phys. Chem. A*, **112**, 1040–1053. 10.1021/jp709896w 18197648

[bb8] Choi, Y.-J. & Jhi, S.-H. (2020). *J. Phys. Chem. B*, **124**, 8704–8710. 10.1021/acs.jpcb.0c05075 32910653

[bb9] Deringer, V. L., Bartók, A. P., Bernstein, N., Wilkins, D. M., Ceriotti, M. & Csányi, G. (2021). *Chem. Rev.* **121**, 10073–10141. 10.1021/acs.chemrev.1c00022 PMC8391963 34398616

[bb10] Deringer, V. L., Caro, M. A. & Csányi, G. (2019). *Adv. Mater.* **31**, 1902765. 10.1002/adma.201902765 31486179

[bb11] Drautz, R. (2019). *Phys. Rev. B*, **99**, 014104.

[bb12] Egami, T. & Billinge, S. J. L. (2012). *Underneath the Bragg peaks: structural analysis of complex materials.* Pergamon.

[bb13] Farmahini, A. H. & Bhatia, S. K. (2015). *Carbon*, **83**, 53–70.

[bb14] Gereben, O., Jóvári, P., Temleitner, L. & Pusztai, L. (2007). *J. Optoelectron. Adv. Mater.* **9**, 3021–3027.

[bb15] Gereben, O. & Pusztai, L. (2012). *J. Comput. Chem.* **33**, 2285–2291. 10.1002/jcc.23058 22782785

[bb16] Hale, L. M., Trautt, Z. T. & Becker, C. A. (2018). *Modell. Simul. Mater. Sci. Eng.* **26**, 055003.

[bb17] Howe, M. A., McGreevy, R. L., Pusztai, L. & Borzsák, I. (1993). *Phys. Chem. Liq.* **25**, 205–241.

[bb18] Hull, S., Norberg, S. T., Ahmed, I., Eriksson, S. G., Marrocchelli, D. & Madden, P. A. (2009). *J. Solid State Chem.* **182**, 2815–2821.

[bb19] Hwang, J., Melgarejo, Z. H., Kalay, Y. E., Kalay, I., Kramer, M. J., Stone, D. S. & Voyles, P. M. (2012). *Phys. Rev. Lett.* **108**, 195505. 10.1103/PhysRevLett.108.195505 23003058

[bb20] Jain, S. K., Pellenq, R. J.-M., Pikunic, J. P. & Gubbins, K. E. (2006). *Langmuir*, **22**, 9942–9948. 10.1021/la053402z 17106983

[bb21] Jinnouchi, R., Miwa, K., Karsai, F., Kresse, G. & Asahi, R. (2020). *J. Phys. Chem. Lett.* **11**, 6946–6955. 10.1021/acs.jpclett.0c01061 32787192

[bb22] Keen, D. A. (2001). *J. Appl. Cryst.* **34**, 172–177.

[bb23] Khadka, R., Baishnab, N., Opletal, G. & Sakidja, R. (2020). *J. Non-Cryst. Solids*, **530**, 119783.

[bb24] Krayzman, V. & Levin, I. (2012). *J. Appl. Cryst.* **45**, 106–112.

[bb25] Krayzman, V., Levin, I., Woicik, J. C., Proffen, Th., Vanderah, T. A. & Tucker, M. G. (2009). *J. Appl. Cryst.* **42**, 867–877.

[bb26] Li, Z., Kermode, J. R. & De Vita, A. (2015). *Phys. Rev. Lett.* **114**, 096405. 10.1103/PhysRevLett.114.096405 25793835

[bb27] Lysogorskiy, Y., Bochkarev, A., Mrovec, M. & Drautz, R. (2023). *Phys. Rev. Mater.* **7**, 043801.

[bb28] Maldonis, J. J., Hwang, J. & Voyles, P. M. (2017). *Comput. Phys. Commun.* **213**, 217–222.

[bb29] McCall, J. (2005). *J. Comput. Appl. Math.* **184**, 205–222.

[bb30] McGreevy, R. L. & Howe, M. A. (1991). *Phys. Chem. Liq.* **24**, 1–12.

[bb31] McGreevy, R. L., Howe, M. A., Nield, V. M., Wicks, J. D. & Keen, D. A. (1992). *Physica B*, **180–181**, 801–804.

[bb32] McGreevy, R. L. & Pusztai, L. (1988). *Mol. Simul.* **1**, 359–367.

[bb33] Metropolis, N., Rosenbluth, A. W., Rosenbluth, M. N., Teller, A. H. & Teller, E. (1953). *J. Chem. Phys.* **21**, 1087–1092.

[bb34] Miksch, A. M., Morawietz, T., Kästner, J., Urban, A. & Artrith, N. (2021). *Mach. Learn. Sci. Technol.* **2**, 031001.

[bb35] Murgida, G. E., Ferrari, V., Ganduglia-Pirovano, M. V. & Llois, A. M. (2014). *Phys. Rev. B*, **90**, 115120.

[bb36] Ohio Supercomputer Center (2018). *Pitzer*, https://osc.edu/ark:/19495/hpc56htp.

[bb37] Opletal, G., Petersen, T. C., O’Malley, B., Snook, I. K., McCulloch, D. G. & Yarovsky, I. (2008). *Comput. Phys. Commun.* **178**, 777–787.

[bb38] Opletal, G., Petersen, T. C. & Russo, S. P. (2014). *Comput. Phys. Commun.* **185**, 1854–1855.

[bb39] Pandey, A., Biswas, P. & Drabold, D. A. (2015). *Phys. Rev. B*, **92**, 155205.

[bb40] Panlener, R. J., Blumenthal, R. N. & Garnier, J. E. (1975). *J. Phys. Chem. Solids*, **36**, 1213–1222.

[bb41] Peterson, P. F., Olds, D., McDonnell, M. T. & Page, K. (2021). *J. Appl. Cryst.* **54**, 317–332. 10.1107/S1600576720015630 PMC7941302 33833656

[bb42] Podryabinkin, E. V. & Shapeev, A. V. (2017). *Comput. Mater. Sci.* **140**, 171–180.

[bb43] Podryabinkin, E. V., Tikhonov, E. V., Shapeev, A. V. & Oganov, A. R. (2019). *Phys. Rev. B*, **99**, 064114.

[bb44] Schran, C., Brezina, K. & Marsalek, O. (2020). *J. Chem. Phys.* **153**, 104105. 10.1063/5.0016004 32933264

[bb45] Shapeev, A. V. (2016). *Multiscale Model. Simul.* **14**, 1153–1173.

[bb46] Soper, A. K. (1996). *Chem. Phys.* **202**, 295–306.

[bb47] Tadmor, E. B., Elliott, R. S., Sethna, J. P., Miller, R. E. & Becker, C. A. (2011). *JOM*, **63**, 17.

[bb48] Thompson, A. P., Aktulga, H. M., Berger, R., Bolintineanu, D. S., Brown, W. M., Crozier, P. S., in ’t Veld, P. J., Kohlmeyer, A., Moore, S. G., Nguyen, T. D., Shan, R., Stevens, M. J., Tranchida, J., Trott, C. & Plimpton, S. J. (2022). *Comput. Phys. Commun.* **271**, 108171.

[bb49] Thompson, A. P., Swiler, L. P., Trott, C. R., Foiles, S. M. & Tucker, G. J. (2015). *J. Comput. Phys.* **285**, 316–330.

[bb50] Tucker, M. G., Dove, M. T. & Keen, D. A. (2001). *J. Appl. Cryst.* **34**, 630–638.

[bb51] Tucker, M. G., Keen, D. A., Dove, M. T., Goodwin, A. L. & Hui, Q. (2007). *J. Phys. Condens. Matter*, **19**, 335218–335244. 10.1088/0953-8984/19/33/335218 21694141

[bb52] Vandermause, J., Torrisi, S. B., Batzner, S., Xie, Y., Sun, L., Kolpak, A. M. & Kozinsky, B. (2020). *npj Comput. Mater.* **6**, 20.

[bb53] Vandermause, J., Xie, Y., Lim, J. S., Owen, C. J. & Kozinsky, B. (2022). *Nat. Commun.* **13**, 5183. 10.1038/s41467-022-32294-0 PMC9440250 36055982

[bb54] Xie, Y., Vandermause, J., Sun, L., Cepellotti, A. & Kozinsky, B. (2021). *npj Comput. Mater.* **7**, 40.

[bb55] Zaverkin, V. & Kästner, J. (2021). *Mach. Learn. Sci. Technol.* **2**, 035009.

[bb56] Zhang, L., Han, J., Wang, H., Car, R. & E, W. (2018). *Phys. Rev. Lett.* **120**, 143001. 10.1103/PhysRevLett.120.143001 29694129

[bb57] Zhang, Y., Eremenko, M., Krayzman, V., Tucker, M. G. & Levin, I. (2020). *J. Appl. Cryst.* **53**, 1509–1518.

